# ChatGPT: the new panacea of the academic world

**DOI:** 10.1590/0037-8682-0060-2023

**Published:** 2023-03-06

**Authors:** Lucindo José Quintans-Júnior, Ricardo Queiroz Gurgel, Adriano Antunes de Souza Araújo, Dalmo Correia, Paulo Ricardo Martins-Filho

**Affiliations:** 1 Universidade Federal de Sergipe, Programa de Pós-graduação em Ciências da Saúde, Aracaju, SE, Brasil.; 2 Universidade Federal de Sergipe, Laboratório de Neurociências e Ensaios Farmacológicos, São Cristóvão, SE, Brasil.; 3 Universidade Federal de Sergipe, Departamento de Medicina, Aracaju, SE, Brasil.; 4 Universidade Federal de Sergipe, Laboratório de Ensaios Farmacêuticos e Toxicidade, São Cristóvão, SE, Brasil.; 5 Universidade Federal de Sergipe, Laboratório de Patologia Investigativa, Aracaju, SE, Brasil.

The chat generative pretrained transformer (ChatGPT) is a language generation model released by OpenAI (San Francisco, California) (https://openai.com/blog/chatgpt/) in November 2022, which is considered a new panacea in academia. This chatbot system is based on neural networks that learn to execute tasks through reading existing human-generated text^1^. Therefore, ChatGPT can produce a wide range of written and unpublished content, such as formal essays and literary, journalistic, and even scientific manuscripts. Remarkably, the texts are often characterized by a high level of originality, coherence between ideas, and furthering of the existing scientific understanding. Moreover, ChatGPT can assist in determining optimal statistical methods for data analysis and transcribing the codes for use in R or Python.

However, the topic is novel and "controversial," and prestigious journals^1^ ^-^ ^5^ are debating the role of ChatGPT and similar systems in the scientific literature, including whether it is appropriate to cite a chatbot as an author^6^ ^,^ ^7^ due to responsibility for the content and integrity of the manuscript. In other words, editors are quickly developing editorial policies for artificial intelligence (AI) tools. Furthermore, the areas of science, technology, and innovation, including the training and qualification of human resources at the *stricto sensu* postgraduate level (masters and doctorates), are based on the generation of new ideas or products; the maturation of currently developed knowledge; or even the training of techniques, procedures, routines, or different types of knowledge for greater fixation, understanding, and, consequently, applicability. Thus, the production of scientific manuscripts, patents, theses, books, and other unique products is common in the academic environment, and copyright issues are invariably required. As scientific integrity is one of the fundamental pillars of the academic world, copying texts from sources without citing them is unacceptable.

AI and its resources are valuable in science, but they cannot replace the researcher's critical and reflective thinking, or their ability to interpret results, discuss their findings based on the best available evidence, and communicate with readers. ChatGPT relies on pre-existing content and lacks the analytical capabilities of humans, such as the ability to weigh values and draw on sensory experiences to make technical and scientific decisions in the current context^7^. Despite their importance, researchers must acknowledge that technologies are not infallible, with their creators recognizing various drawbacks such as incorrect or nonsensical answers, biases in the training data, and the production of insecure content (https://openai.com/blog/chatgpt/). Therefore, we need parsimony and rationality while using text-generating AIs^8^, particularly in science, where communicating the best evidence is a fundamental condition for decision-makers. During a disease outbreak, AI can facilitate the spread of "fake news" by producing scientifically sound texts while supporting false, dangerous, and counterproductive hypotheses. Infodemics have been common during the COVID-19 pandemic^9^, and the ChatGPT can function as an automated tool for disinformation campaigns. However, this technology can assist researchers to prepare scientific manuscripts or other technical products that can save lives at an unprecedented rate.

In light of this new paradigm, a few questions need to be answered:

- Who would be in charge of regulating the use of chatbots in science, and how would this be accomplished?
- What criteria would these regulations be based on?
- What would be the non-negotiable premises?
- Who would handle the follow-up tasks?
- How would those who use it maliciously be punished?

Therefore, researchers are recommend to familiarize themselves with ChatGPT, as this tool will inevitably lead to disruptive changes in science. 
